# Tuberculosis at the International Veterinary Congress

**Published:** 1896-06

**Authors:** 


Tuberculosis at the International Veterinary Congress. Professor Bang began with the remark that since the infectiousness of tuberculosis had been generally recognized many intelligent cattle-owners had striven to lessen the mortality caused by the disease by immediate isolation of infected animals, by not using such animals for breeding purposes, by thoroughly disinfecting the cattle-pens, stalls, etc. Although these means have been productive of good effect, yet they had not come up to expectation, inasmuch as latent cases still remained, and the disease constantly reappeared because there was no means of distinguishing the latent cases; a means was finally found through the great discovery of Dr. Koch. That a diagnosis by means of tuberculin reaction can, as a rule, be established with certainty is, according to Bang, no longer doubtful; but there still exists great difference of opinion in regard to the questions: How often and under what circumstances can wrong diagnosis occur, as well as whether there is danger in the use of tuberculin? According to his report at the Hygienic Congress at Budapest in 1884, Bang placed the number of mistaken diagnoses at 9 per cent. as the result of his investigations. According to his later experiments this figure remains about the same, namely, 9.7 per cent. According to the reports published by Professor Eber not long since, the result is even more unfavorable, reaching as high as 13 per cent. The erroneous diagnoses are of two kinds: 1. Animals may react to tuberculin without the veterinarian being able to show tuberculosis at the autopsy. In regard to this point the speaker emphasized the great difficulty sometimes encountered in detecting the minute tubercles, although present, *e.g.*, in the remote lymph glands, especially when the post-mortem is held by some person little skilled or who did not go about the work with the requisite care and exactness. But he himself in three cases, in which typical reaction had taken place, had been able to find absolutely no tuberculous changes. In one there was found some pyelitis and chronic fibrous nephritis. In the organs affected streptococci and other bacteria were found, but tubercle-bacilli were absent. This is the only case that Professor Bang has had under his care in which the reaction was caused by another chronic, non-tuberculous disease, as has often been stated to be the fact in the literature. He called attention further to the fact that animals which suffer from carcinoma or actinomycosis may at the same time be tuberculous, and in tuberculous processes this fact is often lost sight of. When the result of the autopsy is purely negative in animals that have reacted normally, there must always be doubt as to the existence of tuberculosis. 2. Mistaken diagnosis, where tubercle-bacilli are found present, in spite of the fact that no reaction has taken place. According to his experience this is the case in very insignificant or in thoroughly calcified (reduced to chalk) tuberculous centres, often so old and so perfectly calcified that the investigator involuntarily thinks of a healing process. Although the tuberculin reaction cannot be regarded as infallible, and a diagnosis arrived at through it cannot be offered as proof in judicial cases, nevertheless it has *sufficient value*, according to the results recorded, to *serve as the means* of extirpating tuberculosis. It does little harm if a few non-tuberculous small animals, afflicted with other chronic diseases, are isolated in company with tuberculous ones, even if now and then it should happen that an entirely healthy animal is grouped with infected ones. Great injury may result, on the other hand, if, on the removal of healthy animals from infected places, tuberculous animals should be included among them. Besides, Bang adds that, whenever the tubercular process is extensive, the clinical symptoms will certainly show it, whereas if only calcareous tubercles occur at isolated points in the lymph glands, such animals are certainly *for the time being* not dangerous as communicators of infectious matter. If the tubercular process should extend it can be shown by a subsequent inoculation of tuberculin. Of far greater importance is the question whether tuberculin injections cause an exacerbation of the disease, as has been asserted by Professor Hess. According to Bang's observations, on a large scale, it seems that after inoculation with tuberculin acute miliary tuberculosis occurs only exceptionally, and that this is to be feared only in cases of extensive or far advanced tuberculosis; it occurs, however, when no tuberculin has been used, so that there is no proof that it has been caused by inoculation. If the assertions of Hess be correct, then in herds of cattle in which tuberculin inoculation has been repeatedly done, tuberculosis should prove an extremely unfavorable course; but this is not the case, as Bang proved by a number of statistics. In Denmark tuberculin-inoculation has been practised for years on an extensive scale, and its use is still increasing. This would certainly not be the case if by its means the disease was aggravated. On the contrary, the health of the cattle is constantly improving. As has been remaked, only by tuberculin-inoculation are we in a position to determine the extent of the disease; it seems that the latent form of the disease occurs to a large extent among herds in which tuberculosis has been found; the number reaches sometimes even 70 to 80 per cent. As a result of very careful autopsies on such animals it appears that the disease-process consists merely in small tubercles, mostly in the lymph glands, especially the retro-pharyngeal, bronchial, mesenteric, and mediastinal. Bang gave further the result of tuberculin-injection in 46,495 animals in 1675 herds. The number of animals that reacted amounted to 18,399, or 39.6 per cent., while in 28,096, or 60.4 per cent., no reaction followed. The reaction was very different in the different provinces, depending upon the fact whether cattle traffic was common or whether the herds increased through breeding. In 1170 herds all the animals were inoculated, the number of animals was 30,617. In 93 herds the average number of animals was over 50, in 1077 less than that number. In the larger herds the number of animals that reacted was 59.6 per cent., in the small herds only 33.1 per cent. In the whole number 255 herds were free from tuberculosis; *i.e.*, 252 small herds, but only 3 large herds. In regard to the ages of the animals he noted a great difference in the time of appearance of the tuberculosis. The older the animals the greater the number which reacted. In animals six months old it was only 15.7 per cent., in those one year old 30.3 per cent., in two-year olds 40.6 per cent., and in full-grown animals 50 per cent. According to Bang this is a proof that the disease, as a rule, is spread by infection and is not hereditary. In his opinion one of the principal causes of the disease lies in the use of milk from tuberculous cows.—*From the report of the International Veterinary Congress in the January number of "Tijdschrift voor Veeartsenijkunde."*

